# Natural humoral immune response to ribosomal P0 protein in colorectal cancer patients

**DOI:** 10.1186/s12967-015-0455-7

**Published:** 2015-03-28

**Authors:** Monica Benvenuto, Pierpaolo Sileri, Piero Rossi, Laura Masuelli, Massimo Fantini, Monica Nanni, Luana Franceschilli, Giuseppe Sconocchia, Giulia Lanzilli, Roberto Arriga, Giovanni Faggioni, Florigio Lista, Augusto Orlandi, Vittorio Manzari, Achille Lucio Gaspari, Andrea Modesti, Roberto Bei

**Affiliations:** Department of Clinical Sciences and Translational Medicine, University of Rome “Tor Vergata”, Rome, Italy; Department of Experimental Medicine and Surgery, University of Rome “Tor Vergata”, Rome, Italy; Department of Experimental Medicine, University of Rome “Sapienza”, Rome, Italy; Laboratory of Tumor Immunology and Immunotherapy, Institute of Translational Pharmacology, Department of Medicine, CNR, Rome, Italy; Department of Systems Medicine, University of Rome “Tor Vergata”, Rome, Italy; Centro Studi e Ricerche Sanità e Veterinaria Esercito, Rome, Italy; Department of Biomedicine and Prevention, University of Rome “Tor Vergata”, Rome, Italy

**Keywords:** Ribosomal P proteins, Colon cancer, Immune response, Antibodies

## Abstract

**Background:**

Tumor associated antigens are useful in colorectal cancer (CRC) management. The ribosomal P proteins (P0, P1, P2) play an important role in protein synthesis and tumor formation. The immunogenicity of the ribosomal P0 protein in head and neck, in breast and prostate cancer patients and the overexpression of the carboxyl-terminal P0 epitope (C-22 P0) in some tumors were reported.

**Methods:**

Sera from 72 colorectal tumor patients (67 malignant and 5 benign tumors) were compared with 73 healthy donor sera for the presence of antibodies to CEA, EGFR, ErbB2 and ribosomal P proteins by western blotting or ELISA. Expression of the C-22 P0 epitope on tissues and colon cancer cells was determined by immunoperoxidase staining and indirect immunofluorescence/western blotting, respectively, employing MAb 2B2. Biological effects of MAb 2B2 on colon cancer cells were assessed by the Sulforhodamine B cell proliferation assay, trypan blue exclusion test and cleaved caspase-3 detection. Fisher’s exact test was used to compare the number of auto-antibodies positive patients with healthy donors. Variation in the C-22 P0 expression, and in the number of apoptotic cells was evaluated by Student’s *t*-test. Variation in cell survival and cell death was evaluated by Newman-Keuls test.

**Results:**

No significant humoral response was observed to CEA, EGFR and ErbB2 in CRC patients. Conversely, 7 out of 67 CRC patient sera reacted to ribosomal P proteins. The prevalence of P proteins auto-antibodies in CRC patients was significant. Five patients showed restricted P0 immunoreactivity, while two patients reacted simultaneously to all P proteins. The C-22 P0 epitope was homogenously expressed both in malignant tumors and the adjacent mucosa, but the intensity of expression was higher in the tumor. Starved colon cancer cells showed a higher C-22 P0 epitope plasma membrane expression compared to control cells. MAb 2B2 inhibited colon cancer cell growth and induced cell death in a dose dependent manner.

**Conclusions:**

Our study shows a spontaneous humoral immune response to ribosomal P0 protein in CRC patients and the inhibition of *in vitro* cancer cell growth after C-22 P0 epitope targeting. The ribosomal P0 protein might be a useful immunological target in CRC patients.

## Background

Colorectal cancer (CRC) is the most common form of cancer among those that affect the gastrointestinal tract and represents the third most frequent cancer in men and the second in women [1,2]. Despite advances in diagnosis and therapy, the survival rate of CRC depends on the stage being 90% for stage I and II, and <11% for stage IV [1,3]. The process of CRC carcinogenesis is a multistep process characterized by mutations of several genes, which lead to an invasive and drug resistant-phenotype of the tumor [4-8]. The identification of molecular and immunological targets is essential for improving diagnostic and therapeutic strategies for CRC management.

Tumor antigens expressed by cancer cells are able to elicit spontaneous immune response in cancer patients [9]. Many auto-antibodies have been proposed as diagnostic or prognostic markers in cancer patients although they recognize self-antigens that are overexpressed in tumors [10-14]. It is worth of note that the repertoire of auto-antibodies found in cancer patients partly covers that detected in patients with autoimmune diseases [9]. In addition, auto-antibodies represent useful serological markers in the diagnosis of the autoimmune disease [9]. The immune identification of self-antigens in cancer patients might have significant repercussions that go beyond the discovery of novel biomarkers, since auto-antibodies found in cancer patients can target crucial molecules involved in the carcinogenesis process [15].

Several tumor associated antigens have been shown to be useful in CRC patients. Among them, the most common is the carcinoembryonic antigen (CEA), a glycosylated membrane-bound protein of 180 kDa expressed in a high percentage of several carcinomas, including colorectal, gastric, pancreatic, non-small cell lung and breast carcinomas. The high level tumor expression of CEA and its release in the blood make CEA suitable for using it as tumor marker, especially in patients with CRC [16,17]. Other CRC tumor markers include circulating RNA, MicroRNA, mutated DNA (APC, K-RAS, p53), aberrant methylated markers, CA 19-9, TPA, CA 72-4 and cytokeratin fragments [18-20]. The expression of the epidermal growth factor receptor (EGFR) and human epidermal growth factor receptor 2 (HER2 or ErbB2) are associated with poor prognosis in sporadic CRC, thus representing two important prognostic markers in addition to be employed for targeted-therapy [21,22].

The ribosomal P proteins (P0, 38 kDa; P1, 19 kDa; P2, 17 kDa) are involved in the formation of the ribosomal stalk of the 60 S ribosomal subunit in eukaryotic cell, in which they regulate protein synthesis [23-27]. Recently, we demonstrated the spontaneous immunogenicity of the ribosomal P0 protein in head and neck, in breast and prostate cancer patients and the overexpression of the carboxyl-terminal epitope of P0 (C-22 P0) in head and neck and breast carcinomas [28-30]**.** The immunodominant C-22 P0 epitope was found to be located within the 22 amino acid C-terminal peptide shared by all three P proteins [31,32]. P0 exists as a free protein in the cytoplasm and on the surface of cancer cells [28,33] and appears to promote tumor formation [34]. Auto-antibodies against P proteins have been identified for the first time in systemic lupus erythematosus (SLE) [35]. It was also found that the mRNA level of the P0 was greater in primary colon carcinoma than in paired adjacent normal colon epithelium [36].

In the present study, we investigated the humoral immune response to ribosomal P proteins, CEA, EGFR and ErbB2 in CRC patients and the expression of the C-22 P0 epitope in colon cancer tissues. We also assessed the C-22 P0 epitope expression in two colon adenocarcinoma cell lines and the *in vitro* effect of a monoclonal antibody (MAb 2B2) which recognizes this epitope on the growth of colon cancer cells.

## Methods

### Cell lines, antibodies and proteins

Colon adenocarcinoma cells (HT29 and SW260) were maintained in RPMI 1640 containing 10% fetal bovine serum, 100 U/ml penicillin and 100 μg/ml streptomycin (complete medium). Cells were grown at 37°C in a humidified incubator with an atmosphere of 5% CO_2_. NIH3T3 cells encoding normal rat Neu (LTR-Neu) have been previously characterized and kindly provided by Dr. Eddi Di Marco (Istituto Tumori di Genova) [37]. NIH3T3 cells transfected with expression vectors for human coding sequences of human ErbB family receptors, including LTR-EGFR and LTR-ErbB2, as well as anti-EGFR and anti-ErbB2 antibodies were previously described and kindly provided by Dr. Matthias Kraus [38]. MAb 2B2 is an IgG2a monoclonal antibody, which recognizes the C-22 P0 epitope [28]. Prokaryotic recombinant proteins (P0, P1, P2 and GST), and method of determining the MAb isotype were previously described [28,29,39]. Protein concentration was determined by Bradford protein assay (Bio-Rad, Hercules, CA, USA) [40]. Carcinoembryonic antigen (CEA) was purchased from Vitro Diagnostic Inc (Littleton, CO). The anti-CEA MAb R4 was previously described [41]. Sulforhodamine B, goat anti-human and anti-mouse IgG peroxidase-conjugated antibodies were purchased from Sigma (Milan, Italy). Goat anti-mouse IgG Alexa fluor-488-conjugated antibody was purchased from Life Technologies™ Molecular Probes (Oregon, USA). Anti-human CD3 and anti-human CD20 antibodies were purchased from Ventana Medical System Inc (Tucson, AZ, USA). The anti-activated caspase-3 polyclonal antibody was purchased from Cell Signalling Technology (MA, USA). The purified mouse IgG2a (kappa) UPC10 was purchased from Cappel/Organon Teknika Corporation (West Chester, PA, USA) and used as control.

### Tissues and sera

Tissues and sera of patients were obtained according to the ethical guidelines of the Policlinico of Tor Vergata “PTV”, Rome. Sera from 72 patients with colorectal tumors (colon carcinoma, n = 39; rectal carcinoma, n = 16; sigmoid carcinoma, n = 5; recto-sigmoid carcinoma, n = 7; colon adenoma, n = 5) were collected and compared with 73 healthy donor sera, collected from blood donors from the University of Rome “Sapienza” transfusion center [24 women (mean age: 45.2 ± 14.4) and 49 men (mean age: 47.2 ± 10.7)]. Sera were obtained after informed consent and kept at -20°C until evaluation. The clinical stage of cancer patients included stage I (n = 15), stage IIa (n = 22), stage IIb (n = 2), stage IIIa (n = 1), stage IIIb (n = 21), stage IIIc (n = 4) and stage IV (n = 2). Tissue specimens from 23 cancer patients were also obtained. Adjacent normal mucosa was informative in 17 specimens.

### Detection of anti-Rib-P antibodies

DRG® Anti-Rib-P ELISA kit (EIA-3582, DRG Instruments GmbH, Germany) was employed for detection of IgG auto-antibodies against ribosomal P proteins (P0, P1, P2). The analysis was performed according to the manufacturers’ instructions. Values of anti-rib-P antibodies above 10 U/ml were above the cut-off and thus were considered positive.

### Western blotting

Electrophoresis of purified recombinant P-GST (P0, P1, P2), GST and CEA proteins (0.5 μg⁄lane) as well as NIH3T3 and NIH-LTR-EGFR and LTR-ErbB2 cell extracts (100 μg/lane) was carried out in denaturing 10-12% SDS polyacrylamide gels. Following electrophoresis, proteins were transferred to nitrocellulose membranes at 40 V for 1 h. After blocking in a washing solution (1% Tween-20 in PBS, pH 7.6) containing 5% non-fat dry milk, membranes were incubated overnight at 4°C with either human sera or specific monoclonal and polyclonal antibodies. Human sera were initially titrated at 1:25, 1:50 and 1:100 dilutions [30,42]. The 1:100 dilution was chosen for further experiments since it was the highest serum concentration lacking background reactivity. After extensive washings, membranes were incubated with goat anti-human IgG or goat anti-mouse or anti-rabbit IgG peroxidase-conjugated antibodies. The immune complexes were visualized by the Supersignal West Pico chemiluminescence kit (Pierce, Rockford, IL, USA) [43]. Criteria of serum positivity toward a given antigen consisted in the appearance of an immunoreactive band co-migrating with that detected by the positive control antibody. The intensity of coloring of the specific immunoreactive bands was expressed as densitometric unit (s) (DU) and was obtained using the NIH Pro-Image 1.5 software after blot scanning.

### Immunohistochemical analysis

Expression of the C-22 P0 epitope on tissues was determined by immunoperoxidase staining after incubation with MAb 2B2 or UPC10 (1 μg ⁄mL) as previously described [28,29]. No reactivity was observed using UPC10 (data not shown). Semiquantitative C-22 P0 epitope expression in human tissues was estimated at x200 magnification in at least 10 fields by two investigators in a blind fashion, who used a previously described score system with minor modifications [44,45]. C-22 P0 expression levels (negative, 0; weakly positive, 1; moderately positive, 2; strongly positive, 3) were scored. The interobserver reproducibility was >95%. Paraffin sections were also processed for anti-CD3 and anti-CD20 antibodies analyses using a Ventana XT automated slide stainer (Ventana Medical Sistem Inc, Roche, Tucson AZ, USA) according to manufacturer instruction [46,47]. The ratio between anti-CD3+ and anti-CD20+ cells was evaluated.

### Immunofluorescence staining of the C-22 P0 epitope on colon cancer cell lines (SW260, HT29)

Indirect immunofluorescence was carried out on native cells. Briefly, cells (5 × 10^4^) were grown for 48 hours with or without 10% serum. Cells were detached by incubation with 0.02% EDTA in PBS and incubated with MAb 2B2 or UPC10 (1 μg/ml) for 1 hour at room temperature [48]. After washes with cold PBS, cells were labeled with goat anti-mouse IgG Alexa fluor-488-conjugated antibody (Life Technologies™ Molecular Probes, Oregon, USA) for 45 minutes at room temperature, washed and immediately observed with an Olympus BX51 microscope.

### Sulforhodamine B (SRB) assay

For cell proliferation assay, SW260 and HT29 cells (1 × 10^4^ cells/well) were incubated in serum-free RPMI containing 0.2% BSA containing MAb 2B2 (20, 5, 1 μg/ml) or UPC10 (20 μg/ml). MAbs were replenished every 24 h. All treatments were performed in triplicate. Survival of cells was assessed by the Sulforhodamine B cell proliferation assay after 72 hours, as previously described [49]. The percentage survival of the cultures treated with MAb 2B2 was calculated by normalization of their O.D. values to those of control cultures treated with UPC10 [50].

### Trypan blue exclusion test

For trypan blue exclusion test, SW260 and HT29 cells (5 × 10^4^cells/well) were seeded in 24-well plates. After 24 hours, cells were incubated in serum-free RPMI containing 0.2% BSA and MAb 2B2 (20, 5, 1 μg/ml) or UPC10 (20 μg/ml). Antibodies were replenished every 24 hours. All treatments were performed in triplicate. After 72 hours, adherent as well as suspended cells of each well were harvested and stained with trypan blue (Sigma, Milan, Italy) and counted with an optic microscope. The experiments were repeated three times. Percentage of cell death was determined compared to the total number of cells.

### *In situ* detection of apoptosis

For *in situ* detection of programmed cell death, SW260 and HT29 cells were seeded at 5 × 10^4^ cells/well in 8-well chamber-slides. After 24 hours cells were incubated in serum-free RPMI containing 0.2% BSA and MAb 2B2 (20 μg/ml) or UPC10 (20 μg/ml). Antibodies were replenished every 24 hours. After 48 hours, cells were fixed in 4% paraformaldehyde for 15 min, washed and incubated with the anti-activated caspase-3 polyclonal antibody for 1 hour. After additional washings the cells were labeled with a goat anti-rabbit IgG Alexa fluor-594-conjugated antibody for 30 min [51]. After a third washing, cells were then incubated with 0.1 μg/ml Hoechst 33342 and mounted under a coverslip with glycerol. Cells treated for 16 hours with staurosporine 1 μM were used as positive control. The percentage of apoptotic cells was calculated by determining the ratio between the cells positive for activated caspase-3 and the total number of cells present in five randomly chosen microscopic fields. Cell counts were done in a blinded fashion.

### Statistical analysis

Fisher’s exact test was used to compare the number of patients with positive levels of antibodies compared to healthy donors. Variation in the C-22 P0 expression and in the number of apoptotic cells was evaluated by Student’s *t*-test. Statistical associations were considered significant at a p-value ≤ 0.05.

Data distribution of cell survival and cell death was preliminarily verified by the Kolmogorov-Smirnov test and data sets were analyzed by one-way analysis of variance (ANOVA) followed by Newman-Keuls test. Values with p ≤ 0.05 were considered significant.

## Results

### Immunoreactivity of colorectal cancer (CRC) patient sera to ribosomal P proteins, CEA, EGFR and ErbB2

**Figure 1:**
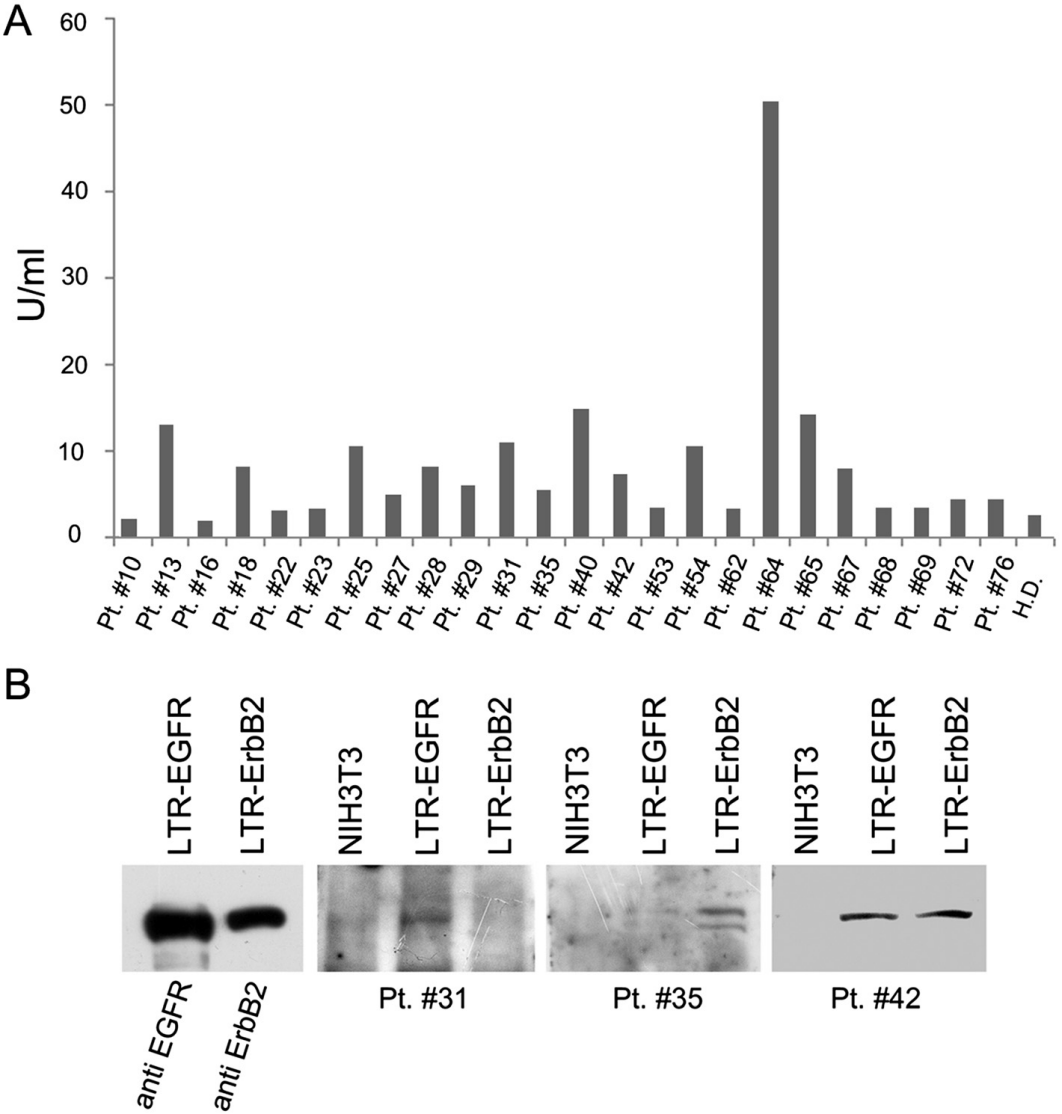
**Humoral immune response to ribosomal P proteins, EGFR and ErbB2 in colorectal cancer patients.** Analysis of the humoral immune response to ribosomal P proteins employing the Rib-P Ab ELISA kit (Panel **A**) and to EGFR and ErbB2 by western blotting employing NIH3T3 cells coding for EGFR (LTR-EGFR) and ErbB2 (LTR-ErbB2) (Panel **B**). Pt. = patient; H.D. = healthy donor.

Sera from colorectal cancer (n = 67) and colon benign tumor patients (n = 5) were analyzed for the presence of auto-antibodies to ribosomal P proteins (P0/P1/P2), CEA, EGFR and ErbB2. The clinicopathological characteristics of patients are reported in Table 1. Patients serum reactivity was compared to that of healthy donors (n = 73). To detect antibodies to ribosomal P proteins, the anti-Rib-P Ab ELISA kit was employed. The presence of serum antibodies to CEA and EGFR or ErbB2 was assessed by western blotting, employing CEA extracted from tumors, and NIH3T3 cells transfected with expression vectors encoding for sequences of human EGFR and ErbB2, respectively. Representative experiments are shown in Figure 1.

**Table 1:** **Clinicopathological characteristics of patients**

**Patients (number)**	**72**
**Age (years)**	
Media	71
Range	43-87
**Gender**	
Male (number)	42
Female (number)	30
**Tumor**	
Adenoma	5
Colon carcinoma	39
Rectal carcinoma	16
Sigmoid carcinoma	5
Recto-sigmoid carcinoma	7
**Stage of disease**	
I	15
IIA	22
IIB	2
IIIA	1
IIIB	21
IIIC	4
IV	2

None of the sera from healthy donors displayed reactivity to P proteins (Table 2). Conversely, 7 out of 67 sera from CRC patients reacted to ribosomal P proteins. The prevalence of anti-P proteins auto-antibodies in CRC patients was significant compared to that of healthy donors (p = 0.0048) (Table 2). The antibody response to anti-P proteins was not associated with the stage of disease. Indeed, patients with anti-P proteins antibodies showed either an early (T1N0, T2N0) or an advanced stage [T3N0 (n = 2) and 3 patients with T3N1 (n = 3)] of disease. In addition, no antibody response to ribosomal P proteins was observed in patients with adenoma.

**Table 2:** **Humoral immune response to ribosomal P proteins, CEA, EGFR and ErbB2 in colorectal cancer patients**

**Subjects**	**Rib-P proteins (P0/P1/P2)**	**p**	**CEA**	**p**	**EGFR**	**p**	**ErbB2**	**p**
**Healthy Donors**	0/73	n.s^a^	0/73	n.s	0/73	n.s	0/73	n.s
**Patients: Benign tumor**	0/5	n.s	0/5	n.s	0/5	n.s	1/5	n.s
**Patients: Malignant tumor**	7/67	0.0048^b^	0/67	n.s	2/67	n.s	1/67	n.s

^a^n.s = not significant.

^b^Patients with malignant tumors vs Healthy donors, Fisher’s exact test.

Conversely, no humoral immune response to CEA was observed in both colorectal cancer patients and healthy donors (data not shown). The anti-CEA MAb R4 was employed as a positive control for CEA detection (Table 2).

Two of 67 cancer patients showed antibodies against EGFR (Table 2). It is important to note that one of these two patients also had antibodies against ribosomal P proteins (data not shown). This patient was diagnosed with cancer of the rectum at an early stage of disease (T1N0). None of the sera from healthy donors displayed reactivity to EGFR or ErbB2. Conversely, one of 5 patients with a benign tumor and one of 67 cancer patients had antibodies to ErbB2 (Table 2). One patient with an advanced stage of disease (T3N2b) displayed a simultaneous reactivity to EGFR and ErbB2.

Our results indicate that the ribosomal P proteins are more immunogenic than CEA and ErbB receptors in CRC patients. The humoral response to P proteins and the C-22 P0 epitope tumor expression were then further investigated. In addition, the *in vitro* effect of MAb 2B2 which recognizes the C-22 P0 epitope on growth of colon cancer cells was analyzed.

### Individual or simultaneous serum reactivity to ribosomal proteins in colorectal cancer patients

**Figure 2:**
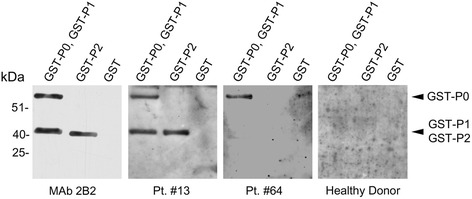
**Humoral immune response to individual ribosomal P proteins.** Analysis of individual ribosomal P proteins was performed by western blotting. The monoclonal antibody (MAb) 2B2 was used as positive control. The protein GST was used as a negative control for the recombinant fusion proteins GST-P0, GST-P1 and GST-P2. Pt. = patient.

The Anti-Rib-P Ab ELISA kit, containing a mixture of the three native P0, P1 and P2 ribosomal proteins, does not allow to identify which P protein is recognized by serum antibodies. To define the individual P protein immunoreactivity of the seven anti-P proteins positive sera, each serum was analyzed for its reactivity to individual recombinant ribosomal P proteins (P0, P1 and P2) by western blotting. Criterion of positivity was the appearance of an immune-reactive band in the serum sample, co-migrating with the one visualized by the MAb 2B2, which recognizes the P0, P1 and P2 proteins. The reactivity of sera to recombinant ribosomal P proteins is shown in Figure 2. The presence of specific antibodies against ribosomal P proteins is shown in Table 3. All seven patients had auto-antibodies to P0 protein. Five patients showed immunoreactivity only to P0, while two patients had simultaneous auto-antibodies to P0, P1 and P2 proteins. No correlation between the concentration of auto-antibodies, detected employing the anti-Rib-P ELISA kit and the density values of immunoreactive bands with sera by western blotting was observed (data not shown).

**Table 3:** **Presence of auto-antibodies directed against ribosomal P proteins**

**Patient #**	**Rib-P assay (U/ml)**	**Western blotting using individual recombinant P proteins**
13	13.21	P0, P1, P2
25	10.62	P0
31	10.93	P0
40	14.74	P0, P1, P2
54	10.56	P0
64	50.25	P0
65	14.12	P0

### Expression of the C-22 P0 epitope in human normal and pathological colorectal tissues

**Figure 3:**
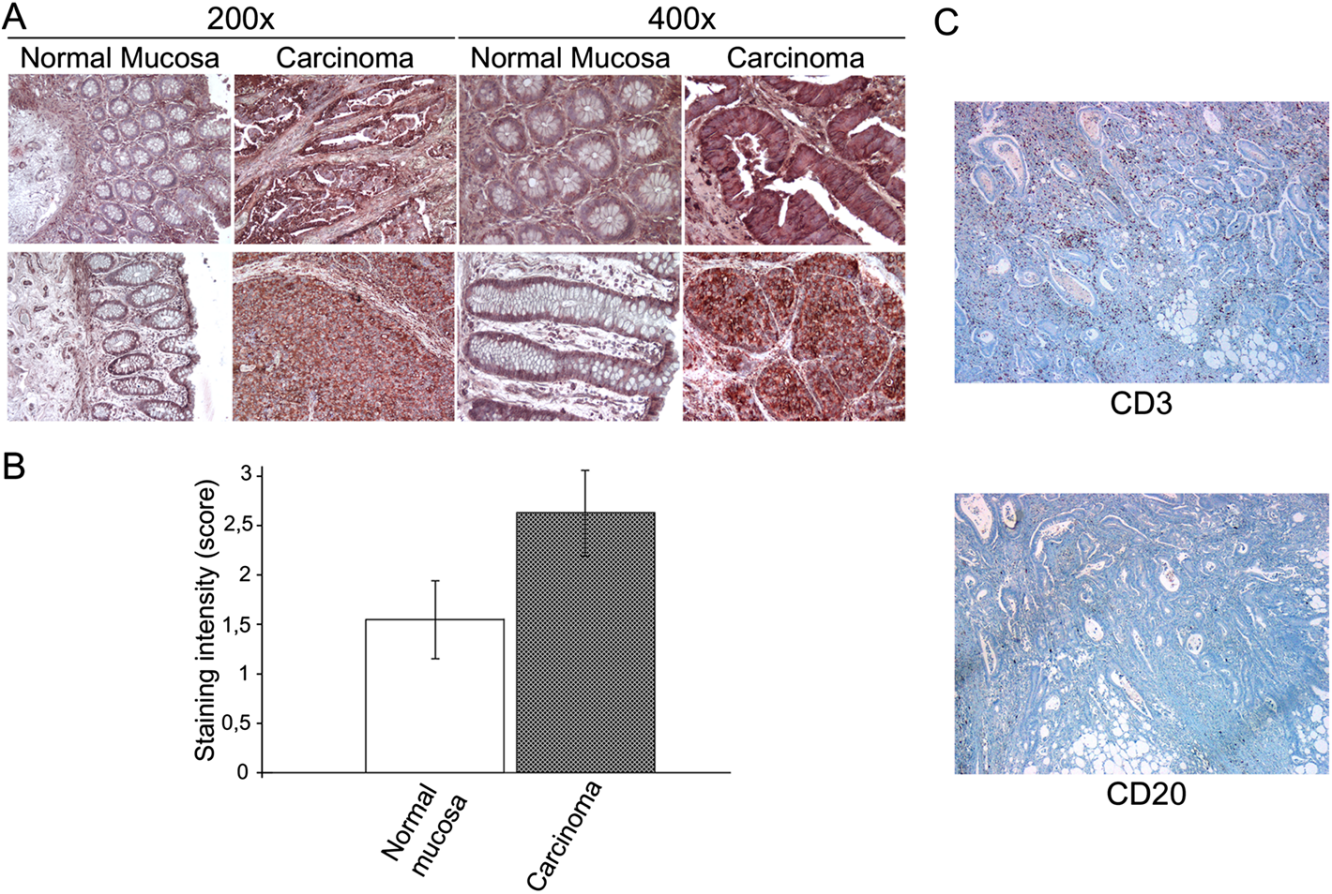
**Expression of the C-22 P0 epitope in normal mucosa and colorectal cancer.** Panel **A**. Immunohistochemical detection of the C-22 P0 epitope by MAb 2B2. Panel **B**. Intensity of the C-22 P0 epitope expression in colorectal tumors and in the adjacent normal mucosa. Bar graph shows a semiquantitative evaluation of the C-22 P0 epitope expression in colorectal tumors and the adjacent normal mucosa (p < 0.001). Panel **C**. Immunohistochemical detection of CD3+ T lymphocytes and CD20+ B lymphocytes in a colon adenocarcinoma from a patient positive for anti-P0 antibodies (40x).

To assess the state of the C-22 P0 epitope expression in tumors, immunohistochemical analysis of colorectal cancer tissues was performed by immunostaining with MAb 2B2 and compared to that of adjacent non-neoplastic mucosa. We evaluated both the intensity and homogeneity of the C-22 P0 epitope expression in 23 malignant tumors and 17 adjacent mucosa samples. Representative immunostaining of the C-22 P0 epitope is showed in Figure 3 (Panel A). The C-22 P0 epitope was expressed homogenously both in the tumor and adjacent normal mucosa, but the intensity of expression was significantly higher in the tumor than in the adjacent mucosa (p < 0.001) (Figure 3, Panel B). It is worth noting that the C-22 P0 epitope was found to be also expressed by fibroblasts, endothelial cells and leukocytes present in the tumor and the normal mucosa.

Tumor specimens from 5 of the 7 patients positive for anti-P0 antibodies were analyzed for the expression of tumor infiltrating T and B lymphocytes. Subserosal deep infiltration of tumor cells was characterized by the presence of abundant inflammatory cells, with a large prevalence of CD3+ T lymphocytes and a small percentage of CD20+ B lymphocytes (mean ratio 5:1) (Figure 3, Panel C).

### Expression and subcellular localization of the C-22 P0 epitope in colon adenocarcinoma cell lines (SW260 and HT29)

**Figure 4:**
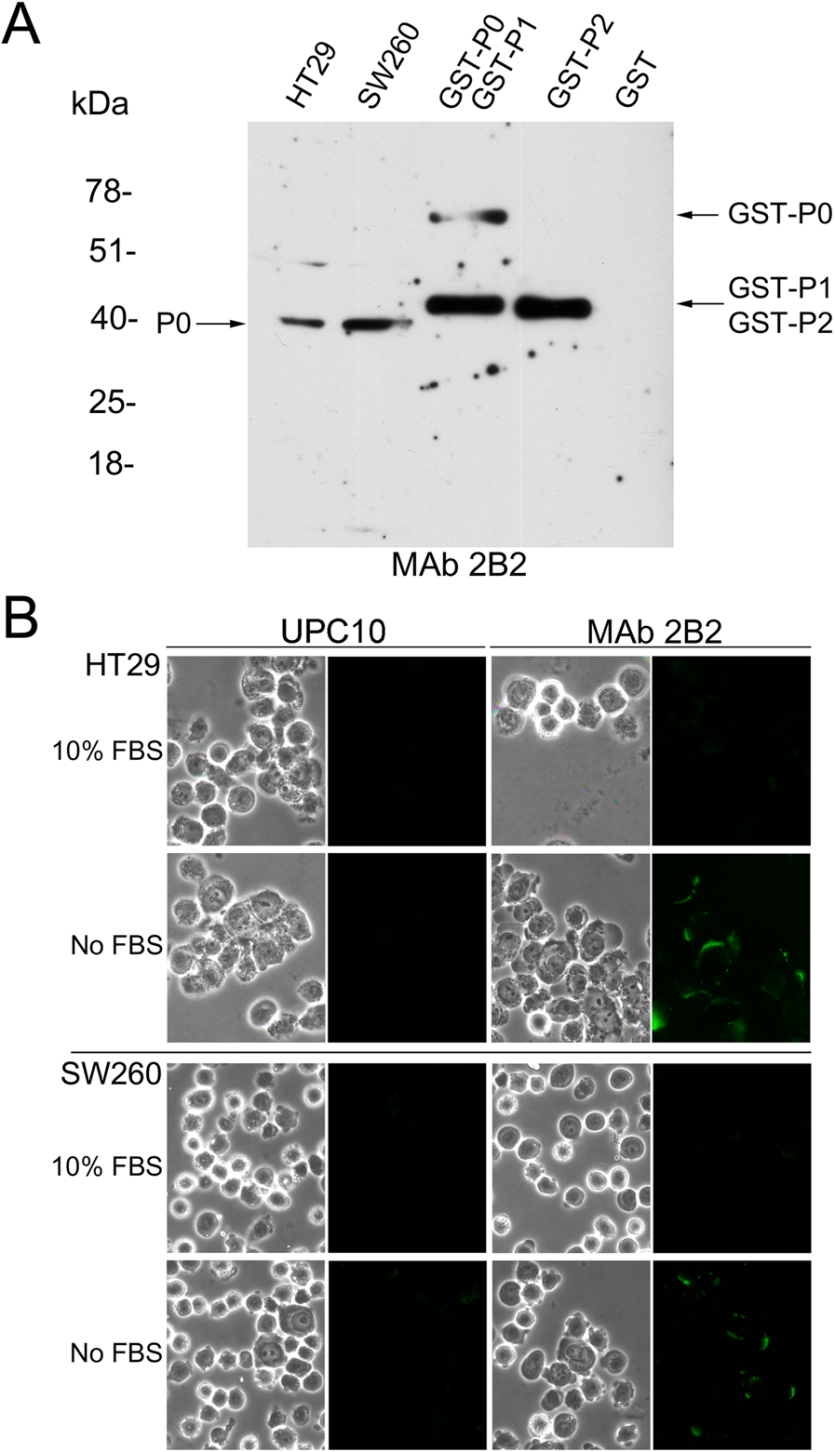
**Expression and subcellular localization of the C-22 P0 epitope in colon adenocarcinoma cell lines (SW260 and HT29).** Panel **A** The expression of the C-22 P0 epitope was determined by western blotting. The recombinant proteins GST-P0/P1/P2 were used as positive control. Panel **B**. Indirect immunofluorescence was performed using not fixed and not permeabilized SW260 and HT29 cells. Cell morphology was determined by phase-contrast microscopy. The antibody UPC 10 was used as a negative control.

The expression of the C-22 P0 epitope on colon adenocarcinoma cells (SW260 and HT29) was analyzed by western blotting. MAb 2B2 detected a molecular weight product of 38 kDa consistent with the molecular weight of the ribosomal P0 protein on both cell lines (Figure 4, Panel A).

We previously demonstrated that the C-22 P0 epitope is localized on the plasma membrane of not fixed and not permeabilized tongue and pharynx cancer cells under conditions of stress, such as the absence of serum in the culture medium [28]. To determine whether the serum deprivation induced a similar effect on colon cancer cells, SW260 and HT29 cells were grown in the presence and absence of serum and indirect immunofluorescence was carried out employing MAb 2B2. As shown in Figure 4, Panel B, SW260 and HT29 cells grown without serum, have a higher expression of the C-22 P0 epitope on the plasma membrane compared to cells cultured in the presence of serum.

### Biological effects of MAb 2B2 on colon adenocarcinoma cells

As shown above, cells under conditions of stress showed increased expression of the C-22 P0 epitope on the plasma membrane. Therefore, we examined whether MAb 2B2 recognizing this epitope was able to inhibit the growth of colon adenocarcinoma cells. For this purpose, SW260 and HT29 colon cancer cells were seeded in serum-free culture medium containing 0.2% BSA and incubated with MAb 2B2 at different concentrations (20, 5 and 1 μg/ml). The antibody UPC10 at 20 μg/ml was used as matched isotype control antibody.

**Figure 5:**
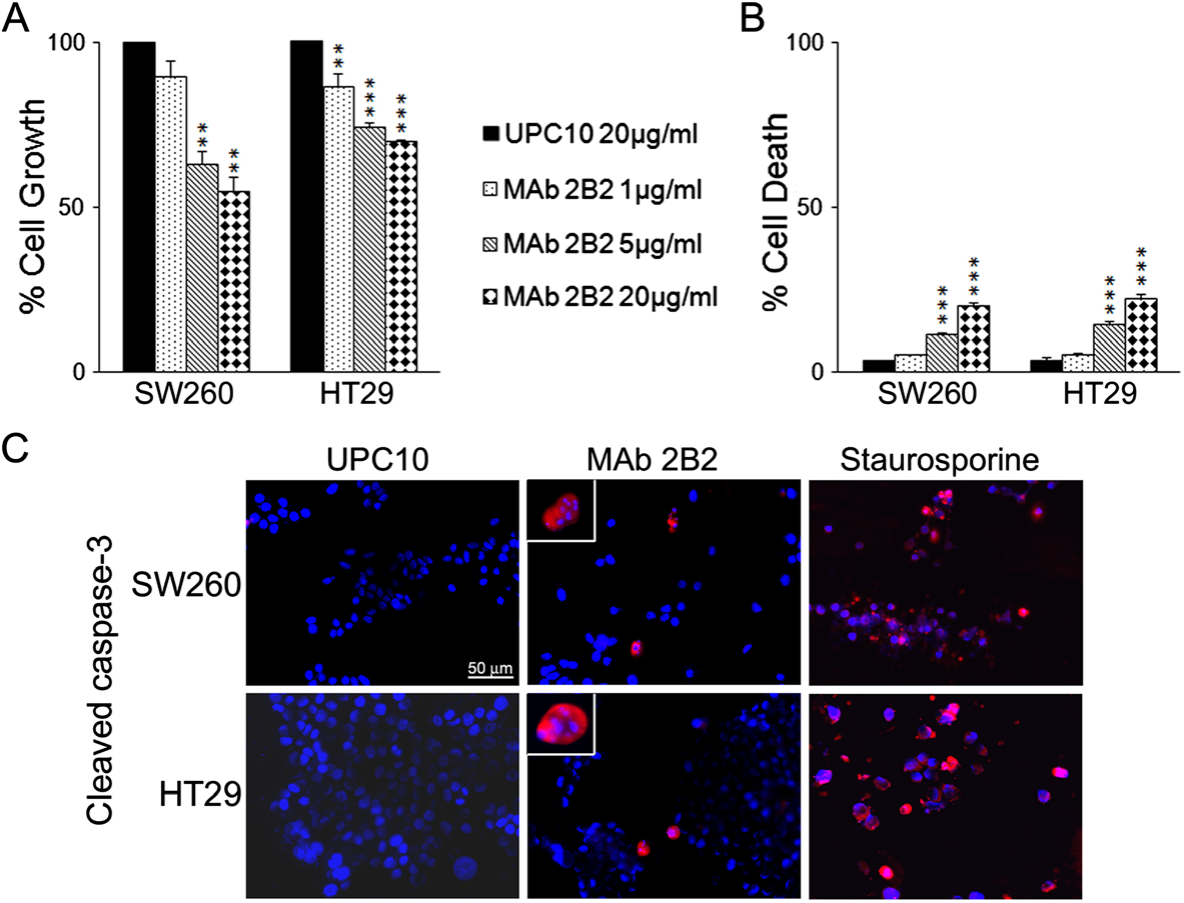
**Effect of MAb 2B2 recognizing the C-22 P0 epitope on colon adenocarcinoma cells.** Panel **A**. Cells growth was assessed by sulforhodamine B assay. SW260 and HT29 cells were treated with MAb 2B2 at different concentrations (20, 5, 1 μg/ml). The antibody UPC10 was used as control (20 μg/ml). The results are the mean of three independent experiments (**p < 0.01, ***p < 0.001). Panel **B**. Trypan blue exclusion test was performed to determine the percentage of cell death of SW260 and HT29 cells treated with MAb 2B2 at different concentrations (20, 5, 1 μg/ml) or with the antibody UPC10 (20 μg/ml). The results are the mean of three independent experiments (***p < 0.001). Panel **C**. *In situ* detection of apoptosis. Induction of apoptosis in SW260 and HT29 cells, as assessed by immunolabeling with an anti-activated caspase-3 antibody, after treatment with MAb 2B2 (20 μg/ml) or UPC10 (20 μg/ml) for 48 hours or, as positive control, with staurosporine (1 μM) for 16 hours. Nuclei were counterstained with Hoechst.

MAb 2B2 significantly inhibited cell growth in both cell lines in a dose dependent manner compared to control antibody. When used at higher concentration (20 μg/ml), MAb 2B2 decreased cell growth of 45% in SW260 cells and 30% in HT29 cells. The mean of the results of three independent experiments is reported in Figure 5, Panel A.

A trypan blue exclusion test and *in situ* detection of apoptosis were carried out in order to evaluate the effect of MAb 2B2 on cell death of colon carcinoma cells. MAb 2B2 significantly increased the percentage of cell death in a dose dependent manner as compared to control antibody (UPC10 at 20 μg/ml) (Figure 5, Panel B). The percentage of cell death upon MAb 2B2 treatment was 11.2% (5 μg/ml) and 19.6% (20 μg/ml) in SW260 cells and 14.1% (5 μg/ml) and 21.9% (20 μg/ml) in HT29 cells.

SW260 and HT29 cells were labeled with an anti-activated-caspase-3 antibody after treatment with MAb 2B2 (20 μg/ml) or UPC10 (20 μg/ml) for 48 hours or, as positive control, with staurosporine (1 μM) for 16 hours. Figure 5, Panel C shows representative pictures of immunolabeled SW260 and HT29 cells. According to activated caspase-3 positivity, the treatment with UPC10 had no relevant effect on the induction of apoptosis in both SW260 (0.3%) and HT29 (0.6%) cells. In comparison, the percentage of apoptotic cells was 6.3% (p < 0.001) and 6.2% (p < 0.001) in MAb 2B2-treated SW260 and HT29, respectively. Treatment with staurosporine resulted in apoptotic rates of 70.1% and 77% in SW260 and HT29, respectively.

## Discussion

One of the most important challenges in the fight against cancer is to find novel biological markers that can help to improve diagnosis and therapy. Several tumor antigens are recognized by the immune system and thus might represent biological markers for the early detection of cancer [52]. The complexity in the diagnosis of CRC might be improved by the discovery of new diagnostic markers [53]. Several tumor markers have been identified in CRC [54]. Recently, it has been evaluated the presence of auto-antibodies to tumor associated antigens as a biological marker. It has been described that auto-antibodies are elicited during the transition from benign neoplasm or chronic inflammatory disease to malignancy [55]. Auto-antibodies to CENP-F and P62 appear to be linked to the transition from chronic liver disease to hepatocellular carcinoma [56]. In our study, we evaluated the humoral immune response to ribosomal P0, P1 and P2 proteins, CEA, EGFR and ErbB2 in CRC patients and compared to that in healthy donors.

Among the antigens analyzed, the ribosomal P0 protein was found to be the most immunogenic antigen in CRC patients. Indeed, patients with CRC showed a significant humoral response to ribosomal P0 protein compared to healthy donors. On the other hand, a significant immune response to CEA, EGFR and ErbB2 was not observed. The spontaneous immunogenicity of the ribosomal P0 protein had already been observed in previous studies performed in our laboratory [28-30]. The analysis of the immune response with head and neck cancer patients showed a significant response against the ribosomal P0 protein. Immunity to P0 protein (7/40) was associated with malignancy and advanced disease stage, but it was not dependent on the C-22 P0 epitope overexpression [28]. In prostate cancer patients, a significant humoral response to P0 protein correlated with the blood release of the prostate specific antigen (PSA) after radiotherapy [30]. However, in CRC patients we could not observe any association between the immune response and the stage of the disease.

Auto-antibodies against P proteins have been identified for the first time in systemic lupus erythematosus (SLE) where they can induce damage of the central nervous system, hepatitis and nephritis. In particular, anti-P proteins antibodies appear to be associated with other forms of SLE with psychosis, where the antibody titer increases before and during the active phase of psychosis [35]. However, patients with CRC displaying P0 protein auto-antibodies, did not show liver or kidney alterations, neither were affected by psychosis. The lack of normal tissue damage by spontaneously elicited anti-P0 antibodies in CRC patients might be due to a low serum auto-antibodies concentration. Patients with SLE might have a higher titer of anti-P0 antibodies compared to cancer patients. In addition, some reports failed to find any relationship of anti-P antibodies and neuropsychiatric SLE or liver and renal diseases in SLE patients [57,58].

In our study, we also demonstrated, by immunohistochemistry employing MAb 2B2, that the CRC tissue shows an overexpression of the C-22 P0 epitope compared to adjacent normal mucosa. The C-22 P0 epitope was found to be overexpressed in head and neck [28], and breast [29] cancers. In addition earlier studies reported high level of P0 messenger RNA in hepatocellular and colon carcinomas [36]. In gynecological tumors, the ribosomal P0 protein expression did not correlate with the stage of disease although the expression increased primarily in the cancerous tissue and not in precancerous lesion [59]. The ribosomal protein P0 appears to promote tumor formation [34]. Indeed, it was observed that it is able of binding the protein GCIP (interacting protein cyclin D1 and GRAP2), which is a tumor suppressor localized in the nucleus, which inhibits the phosphorylation of RB (retinoblastoma protein). In addition, P0 also promotes the increased expression of cyclin D1 and cell proliferation, when it is overexpressed [34].

Different monoclonal antibodies have been approved for the treatment of hematopoietic and solid tumors [60]. MAbs can bind antigens differentially expressed by cancer cells compared to the normal counterparts and block directly or through immune system the growth of cancer cells [60]. Accordingly, we evaluated the C-22 P0 epitope expression on the plasma membrane of colon cancer cells, since we earlier demonstrated that this epitope is expressed on the membrane of tongue and pharynx cancer cells under conditions of stress [28]. We observed that colon cancer cells grown without serum, showed a higher expression of the C-22 P0 epitope on the plasma membrane compared to cells cultured in the presence of serum. It should be considered that solid tumors could grow in an adverse microenvironment characterized by improper vascularization and poor oxygen and nutrient supply [61]. In addition, it was demonstrated that the ribosomal P0 protein is expressed in the membrane in Jurkat cells and T-lymphoblasts, and becomes the target for anti-P0 auto-antibodies, which were internalized and caused cell apoptosis [62]. Anti-P0 antibodies were shown to penetrate into living hepatoma cells and cause cellular dysfunction in culture as well [63,64]. We previously demonstrated that BALB-neuT mice vaccinated with the human P0 protein had a significant delay of neu-mediated mammary carcinoma growth and that the extent of tumor growth interference *in vivo* was associated with high serum levels of antibodies, which recognize the murine P0 protein expressed on mouse mammary cancer cells [29].

Accordingly, we evaluated the biological effect of MAb 2B2 on the *in vitro* growth of cancer cells. Here, we demonstrated that MAb 2B2 significantly inhibited human colon adenocarcinoma cell growth in a dose dependent manner. The inhibition of survival observed in MAb 2B2-treated cells could be due to both reduced proliferation and increased cell death. Therefore, we analyzed the effect of MAb 2B2 on colon cancer cells viability and apoptosis. We demonstrated that MAb 2B2 significantly reduced colon cancer cell viability and induced apoptosis in a dose dependent manner in both colon cancer cell lines.

Overall, our results indicate the potential usefulness of the ribosomal P0 protein as an immunological target in CRC patients, who spontaneously develop auto-antibodies to the P0 protein. Further studies should be performed to study the potential involvement of anti P0 auto-antibodies in cancer cells death. Our study showing for the first time a spontaneous humoral immune response to ribosomal P0 protein in CRC patients and the inhibition of *in vitro* cancer cell growth after C-22 P0 epitope targeting, might offer a potential tool for designing cancer vaccines targeting the P0 protein, in order to enhance the immune response for hampering tumor growth.

## Conclusions

Our study shows for the first time a spontaneous humoral immune response to ribosomal P0 protein in CRC patients and the inhibition of *in vitro* cancer cell growth after C-22 P0 epitope targeting. The ribosomal P0 protein might be a useful immunological target in CRC patients.

## Abbreviations

CEA: Carcinoembryonic antigen
EGFR: Epidermal growth factor receptor
GST: Glutathione S-transferase
